# Novel Approaches for Diagnosing and Management of Cardiovascular Disorders Mediated by Oxidative Stress

**DOI:** 10.1155/2020/7096727

**Published:** 2020-04-20

**Authors:** Adrian Doroszko, Aneta Radziwon-Balicka, Robert Skomro

**Affiliations:** ^1^Department of Internal Medicine, Occupational Diseases and Hypertension, Wroclaw Medical University, Wroclaw, Poland; ^2^Department of Clinical Experimental Research, Glostrup Research Institute, Rigshospitalet, Glostrup, Denmark; ^3^Division of Respirology, Critical Care and Sleep Medicine, Department of Medicine, University of Saskatchewan, Saskatoon, Canada

The cardiovascular disease is one of the major healthcare problems of the world population, and understanding its determinants is essential for designing effective interventions.

Reactive oxygen species (ROS) are toxic, highly reactive, and unstable compounds formed during a variety of physiological and pathological biochemical reactions. ROS are produced in all viable cells, and strong evidence suggests an important role of ROS in the development and progression of cardiovascular disease. Nevertheless, the precise mechanisms contributing to the cardiovascular system injury due to increased oxidative stress are still under investigation. Recent experimental studies suggest that ROS plays a causational role in the development of systolic dysfunction following myocardial infarction (MI), the brain ischemia-reperfusion injury in the course of stroke and in cardiometabolic disorders. Endothelium plays a crucial role in regulation of vascular tone, and endothelial dysfunction (ED) is an important risk factor of cardiovascular disease. The mechanisms involved in decreased vasodilative activity of endothelial cells include decreased bioavailability of nitric oxide, oxidative stress, and disorders in the metabolism of prostanoids. The decreased bioavailability of nitric oxide (NO), as well as increased oxidative stress, plays a crucial role in contributing to the decrease of endothelial vasodilative properties. According to several experimental studies, increased peroxynitrite (ONOO^−^) formation correlates with the development of neurological deficits following ischemic stroke and the cardiac ischemia-reperfusion injury in the course of coronary artery disease and development of peripheral atherosclerotic lesions. Therefore, increased ONOO^−^ production followed by ONOO^−^-dependent protein modifications should be considered as one of the molecular mechanisms contributing vascular injury. The peroxynitrite-dependent modifications of proteins have been shown in many cardiovascular disorders; however, its molecular consequences still remain unknown.

Sufficient synthesis and bioavailability of nitric oxide is crucial for proper functioning of vascular endothelium. Consequently, NO deficiency is prerequisite for and a hallmark of endothelial dysfunction, a pathology preceding the development of cardiovascular disease (CVD). CVD and its main risk factors, such as obesity, hypertension, and type 2 diabetes mellitus (T2DM), are, in turn, among the key factors negatively affecting proper wound healing. Recently, a decreased nitric oxide bioavailability as expressed by an elevation in serum ADMA and decrease in serum L-arginine have been reported in patients with chronic wounds. Detrimental effects of diminished NO bioavailability on cardiovascular health and wound healing are well documented and have led to an outburst of novel treatment strategies aiming at its increase. Therefore, in a study by M. Krzystek-Korpacka et al., a wider panel of the L-arginine/ADMA/NO pathway metabolites using a targeted metabolomics approach was used in order to determine their status and clinical relevance in patients with chronic wounds of various etiologies. The authors demonstrate that patients with chronic wounds in the course of cardiometabolic disorders have reduced bioavailability of NO and its substrate, arginine, resulting from ADMA and SDMA accumulation rather than from arginine deficiency. Citrulline was decreased in patients with cardiometabolic diseases in general, but the presence of chronic wounds is associated with its elevation, reflecting degree of ADMA and SDMA accumulation and inversely related to NO and arginine bioavailability.

E. Romuk and colleagues have evaluated a wide range of oxidative stress markers and their impact on mortality and morbidity in patients with chronic heart failure. Malondialdehyde, a marker of lipid peroxidation, and serum uric acid level were strongly associated with worse prognosis in this group of subjects. The authors postulate that hat validation of elevated malonyldialdehyde and uric acid levels as independent predictors of outcome could have a potentially significant value for risk stratification of chronic HF patients.

Oxidized-LDL-induced inflammation, as a mediator atherosclerosis in malignancies, was investigated by L. Wang et al. Thymocyte selection-associated high mobility group box (TOX), which was reported to be regulated by lncRNA, has been closely related to the immune cell-associated proliferative diseases, such as cancer. Human metastasis-associated lung adenocarcinoma transcript 1 (MALAT1), an 8.7 kb lncRNA, has been demonstrated to be overexpressed in several cancers, but the roles of MALAT1 in the pathogenesis of CVDs are still not well defined. In this study, the authors evaluated the crosstalk between MALAT1 and TOX through investigating whether the regulatory mechanism was associated with the miRNA network. The authors demonstrate that suppression of MALAT1 may attenuate inflammation in oxLDL-incubated endothelial cells by upregulating miR-181b and inhibiting the expression of TOX, which is closely related to the inhibition of the MAPK signaling pathway that attenuate the pathogenesis of atherosclerosis.

Lectin-like oxidized-LDL receptor-1 (LOX-1) is the major receptor for oxidized low-density lipoprotein (oxLDL), and targeting LOX-1 may provide a novel diagnostic strategy towards hypercholesterolemia and vascular diseases. In a study by A. Singh et al., the aegeline was shown to be effective in reducing the lipid abnormalities in aged hypercholesterolemic rats when compared to atorvastatin by targeting LOX-1 and had a pronounced effect in downregulating the expression of oxidized-LDL. Interestingly, T. Wielkoszynski et al. showed that 5*α*,6*α*-epoxy-phytosterols and 5*α*,6*α*-epoxy-cholesterol similarly impair the redox state in rats by increasing the production of free oxygen radicals and free radical-mediated lipid modification, as well as by affecting the mechanisms of nonenzymatic antioxidant defense and the activity of antioxidant enzymes.

A. Stanek et al. in an original study estimated the impact of whole-body cryotherapy (WBC) and subsequent kinesiotherapy on oxidative stress and lipid profile when performed in a closed cryochamber on healthy subjects. Until now, WBC has been used mainly in sports medicine and in the treatment of locomotor system diseases. In the available literature, there has been only one study, which estimated the influence of WBC on lipid profile parameters in healthy subjects, but WBC procedures were not connected with a subsequent session of kinesiotherapy, and the authors only estimated lipid profile parameters. As results from this study, a significant decrease of oxidative stress markers and total cholesterol as well as LDL and a significant increase of total antioxidant capacity were observed following WBC treatment. The activity of plasma SOD-Mn and erythrocyte total SOD increased significantly in the WBC group. On the other hand, M. Bernardi with colleagues investigated the cardiovascular risk factors and hematological indexes of inflammation in Paralympic Athletes with different motor impairment. Interestingly, Paralympic Athletes with lower limb amputation had a higher cardiometabolic risk, whereas Paralympic Athletes with spinal cord injury had a higher platelet-derived cardiovascular risk.

Vascular smooth muscle cells (VSMCs) are the center of the calcification process. Many studies have confirmed that HAP crystals cause damage to VSMCs and induce cell phenotype transformation, which in turn promote vascular calcification. For example, exogenous calcifying nanoparticles, which are nanosized complexes of calcium phosphate mineral and proteins, are taken up by aortic smooth muscle cells in vitro, thereby decreasing cell viability, accumulating apoptotic bodies at mineralization sites, and accelerating vascular calcification. Interestingly, S. Xiong et al. have shown that stimulation of Na/K-ATPase with an antibody against its 4 extracellular region attenuates angiotensin II-induced H9c2 cardiomyocyte hypertrophy via an AMPK/SIRT3/PPAR*γ* signaling pathway. In this study, the effects of the differences in the morphological characteristics of nano-HAP on rat aortic smooth muscle cell (A7R5) injury and its phenotypic transformation were investigated to provide a basis for the determination of the effects of the physicochemical properties of crystals on cellular toxicity and vascular calcification. The extent of cell damage was closely related to the morphological characteristics of the crystals. More calcium deposits on the cell surface, more expressions of osteogenic protein (BMP-2, Runx2, OCN, and ALP), and a stronger osteogenic transformation ability were observed in the crystal with a high cell cytotoxicity than in the other crystals with a low cytotoxicity, thus increasing the risk of vascular calcification.

A very interesting study on calcific aortic valve stenosis (CAVS) was performed by N. Mercier and colleagues. Oxidative stress could be one potential mechanism that increases valve calcification and CAVS disease burden. Semicarbazide-sensitive amine oxidase (SSAO), also known as vascular adhesion protein-1 (VAP-1), is a mediator of tissue oxidative stress and a contributor to atherosclerotic plaque development. Furthermore, serum levels of SSAO are higher in patients with severe CAVS compared with patients presenting moderate CAVS and are significantly correlated with CAVS severity as assessed by echocardiography. The study by N. Mercier et al. is the first report showing a gradual and significant increase in SSAO mRNA, protein, and activity in human aortic valves divided into healthy, intermediate, and calcified tissue. The SSAO upregulation with valve calcification was independent of the cardiovascular and CAVS risk factors obesity, diabetes, and smoking. Furthermore, a significant correlation of SSAO expression with pathways of oxidative stress was also revealed. The results of this study indicate a link between SSAO, oxidative stress, and aortic valve calcification and point to SSAO inhibition as a putative therapeutic approach to be explored for the prevention of valve calcification and CAVS progression.

With respect to aortic stenosis therapeutic strategy (classical surgical valve replacement, SAVR vs. transcatheter valve replacement, TAVR), M. Mahmoudi and colleagues compared the early oxidative stress response in the blood of patients undergoing TAVR with a group of patients undergoing SAVR by applying established biochemical readouts of cellular and extracellular redox status. As compared to patients undergoing SAVR, patients undergoing TAVR did not show significant changes in biomarkers of oxidative stress despite having greater comorbidities and impaired baseline antioxidant defenses. TAVR was associated with an improvement in the antioxidant capacity of plasma.

This special issue provides also some new data on novel therapeutic targets as well as some unknown, additional pleiotropic effects of drugs already used in clinical practice.

High glucose-induced cardiomyocyte injury is the leading cause of diabetic cardiomyopathy, which is associated with the induction of inflammatory responses and oxidative stress. A member of the G protein-coupled receptor family, G protein-coupled bile acid receptor 1 (GPBAR1; also known as TGR5), plays an important role in the regulation of glucose metabolism and has been recently identified as a drug target in type 2 diabetes. TGR5 is activated by bile acids and mediates the endocrine effects of bile acids on energy balance, inflammation, and digestion and regulates insulin secretion to maintain glucose homeostasis. The TGR5 ligand (oleanolic acid) shows significant blood glucose-lowering and weight-losing effects in diabetic animal models. L. Deng and colleagues have shown that activation of TGR5 partially alleviates high glucose-induced cardiomyocyte injury by inhibition of inflammatory responses and oxidative stress and postulate that activation of TGR5 has cardioprotective effects against HG-induced cardiomyocyte injury by suppressing inflammation and apoptosis partially through inhibiting the NF-*κ*B pathway and activating the Nrf2 pathway. Therefore, the authors postulate that TGR5 could be a pharmacological target for the treatment of diabetic cardiomyopathy.

M. Trocha et al. analyzed the antidiabetic SGLT-2 inhibitor-dependent differences in the intensity of oxidative stress in rat livers subjected to ischemia-reperfusion injury. Among the many phenomena occurring in the IR, there is an excessive production of free radicals and the development of oxidative stress. A body of evidence has gathered concerning protective effect of new drugs on hepatic cells during IR, providing rationale for new therapeutic strategies. Among others, glucose-lowering activity of incretins and hence indirectly of sitagliptin, STG, translates into reduced oxidative stress, condition fueled by hyperglycemia (21). Moreover, STG has been found to be an efficient scavenger of reactive oxygen species (ROS), directly reducing superoxide generation in various organs. In this study, a protective effect of SGLT-2 inhibitor on the rat liver, especially its antioxidant properties, was revealed under IR conditions. Also, despite the small degree of steatosis, the aminotransferase activity analysis does not suggest any hepatotoxic action of STG. Contrarily, even a slight protective effect of this drug was seen, especially in IR conditions.

Interestingly, A. Bilska-Wilkosz et al. demonstrated that lipoic acid (LA) may exert beneficial effects on ethanol-induced cardiotoxicity. The administration of ethanol, linoleic acid, and disulfiram separately or jointly affected the aldehyde dehydrogenase activity in the rat liver indicating that LA is an inhibitor of aldehyde dehydrogenase. This study for the first time demonstrated that LA could partially attenuate the cardiac arrhythmia (extrasystoles and atrioventricular blocks) induced by EtOH and reduced the EtOH-induced mortality of animals, which supports a potential of LA for use in acute EtOH-intoxication and suggests that further experiments are necessary to elucidate the mechanism of action of LA as an antidote to EtOH poisoning.

Another original paper, by A. Barbosa et al., points out that the redox-active drug, MnTE-2-PyP5+, prevents and treats cardiac arrhythmias preserving the cardiac systolic function.

The death of cancer survivors was mainly attributed to cardiac factors, which emphasizes the need for pharmacological strategies offering protection against cardiotoxicity caused by anticancer drugs. Doxorubicin (DOX), an anthracycline chemotherapeutic, has been widely used for the treatment of both solid and hematologic malignancies, but its therapeutic use is limited by its dose-dependent cardiotoxicity, resulting in the cardiomyocyte loss, mitochondrial dysfunction, myofibrillar degeneration, and congestive heart failure with poor prognosis. In the study by X. Hu et al., the authors demonstrate that miR-200a supplementation, by activating Nrf2, could reduce cardiac injury, improve cardiac function, and attenuate DOX-related oxidative stress and cell apoptosis. miR-200a also protected the hearts from DOX-induced chronic damage and may thus, according to the authors, represent a new cardioprotective strategy against DOX-induced cardiotoxicity.

